# Effect of sildenafil added to antifibrotic treatment in idiopathic pulmonary fibrosis

**DOI:** 10.1038/s41598-021-97396-z

**Published:** 2021-09-08

**Authors:** Jieun Kang, Jin Woo Song

**Affiliations:** 1 Division of Pulmonary and Critical Care Medicine, Department of Internal Medicine, Ilsan Paik Hospital, Inje University College of Medicine, Goyang-si, Gyeonggi-do Republic of Korea; 2 Department of Pulmonary and Critical Care Medicine, Asan Medical Center, University of Ulsan College of Medicine, 88 Olympic-Ro 43-gil, Songpa-gu, Seoul, 05505 Republic of Korea

**Keywords:** Respiratory tract diseases, Outcomes research

## Abstract

Sildenafil is a phosphodiesterase-5 inhibitor used to treat idiopathic pulmonary arterial hypertension; however, its benefits are unclear in patients with advanced idiopathic pulmonary fibrosis (IPF). We aimed to evaluate its effect as an add-on to antifibrotic agents on clinical outcomes of real-world IPF patients. Among a total of 607 IPF patients treated with antifibrotic agent, 66 concurrently received sildenafil. Propensity score matching was performed to adjust for differences in age, sex, body mass index, forced vital capacity (FVC), and diffusing capacity (DL_CO_) between the sildenafil and no-sildenafil groups. The outcomes of these groups in terms of FVC decline rate, all-cause mortality, hospitalization, and acute exacerbation were compared. Propensity score matching identified 51 matched pairs. The mean age of the patients was 69.5 years and 80.4% were male. Mean FVC and DL_CO_ were 51.7% and 29.5% of the predicted values, respectively. The FVC decline rates did not differ significantly (*p* = 0.714) between the sildenafil (− 101 mL/year) and no-sildenafil (− 117 mL/year) groups. In multivariable analyses adjusted for comorbidities and presence of pulmonary hypertension, sildenafil had no significant impact on all-cause mortality, hospitalization, or acute exacerbation. Sildenafil add-on to antifibrotic treatment had no significant effects on the clinical outcomes of IPF patients.

## Introduction

Idiopathic pulmonary fibrosis (IPF) is a fatal progressive fibrosing interstitial lung disease characterized by worsening dyspnea and lung function^1^. Two antifibrotic medications, pirfenidone and nintedanib, have been shown to delay progression^2,3^, but they are not able to cure or reverse the disease. The fibrotic destruction in IPF involves both the lung parenchyma and pulmonary vasculature^4,5^. Previous studies have shown vascular abnormalities in IPF, including hypoxic vasoconstriction, ablation of vessels in areas of severe fibrosis, and aberrant microvascular and macrovascular remodeling, which ultimately lead to pulmonary hypertension^6,7^.

Sildenafil is a phosphodiesterase-5 inhibitor used to treat idiopathic pulmonary arterial hypertension^8^. Previous studies have evaluated whether sildenafil is also effective in patients with IPF. A randomized controlled trial conducted in 180 patients with advanced IPF, defined as a carbon monoxide diffusion capacity (DL_CO_) < 35% predicted, found promising effects of sildenafil in terms of some secondary endpoints, including quality of life, degree of dyspnea, and arterial oxygenation^9^. In addition, the benefits of sildenafil were more prominent in patients with right ventricular (RV) dysfunction^10^. These results suggested that sildenafil might be helpful in advanced IPF, which is often accompanied by pulmonary hypertension.

Sildenafil has been studied more recently in conjunction with nintedanib or pirfenidone^11,12^. Unfortunately, these studies failed to find statistically significant benefits in primary outcomes. Moreover, the role of sildenafil is difficult to define, given the studies’ conflicting results. In a prespecified subgroup analysis of the INSTAGE trial, the decline in the forced vital capacity (FVC) of the patients on nintedanib alone was numerically greater than in those taking nintedanib plus sildenafil, regardless of the presence of right heart dysfunction^13^. In contrast, a recent phase 2b randomized trial showed that sildenafil plus pirfenidone was associated with a greater decline in FVC than pirfenidone alone^11^. The benefits, if any, of sildenafil add-on treatment to antifibrotic agents in terms of clinical outcomes such as mortality, hospitalization, and acute exacerbation are not clear. In this study, we aimed to evaluate the effect of sildenafil add-on to antifibrotic treatment on the clinical outcomes of real-world IPF patients.

## Methods

### Study patients

**Figure 1:**
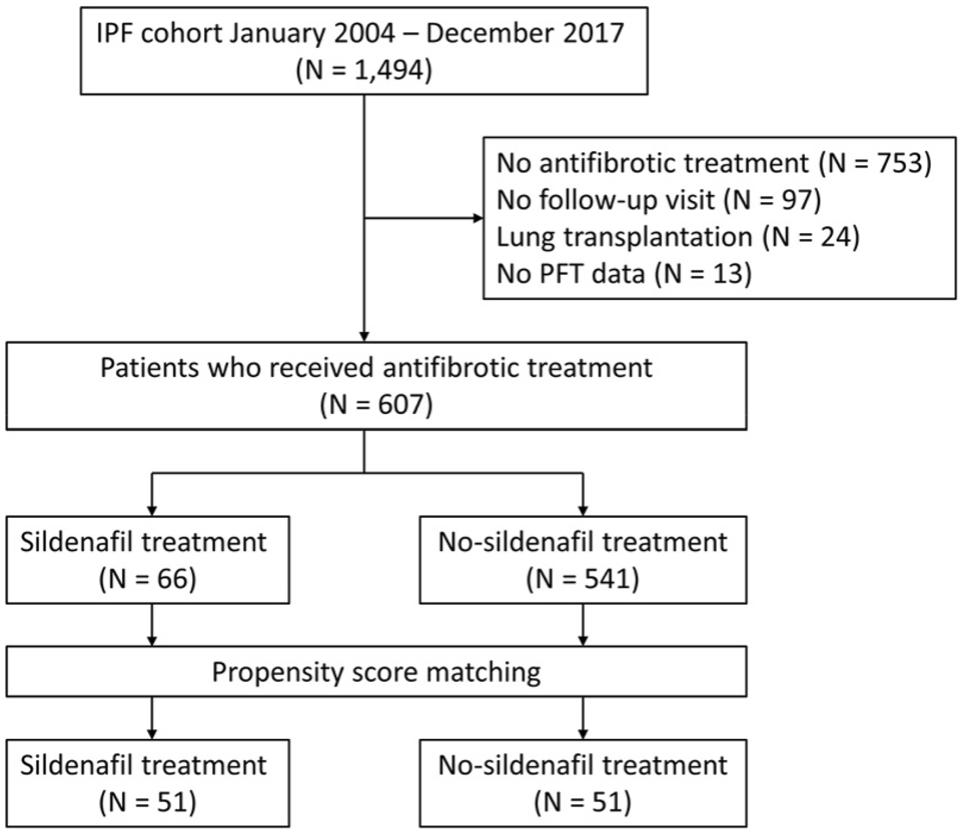
Study flow. IPF, idiopathic pulmonary fibrosis; PFT, pulmonary function test.

We identified 1,494 patients diagnosed with IPF at Asan Medical Center, Seoul, Republic of Korea, between January 2004 and December 2017. Among them, patients were excluded if they (1) did not receive antifibrotic treatment (n = 753), (2) did not have any follow-up visit (n = 97), (3) underwent lung transplantation (n = 24), or (4) did not have pulmonary function data (n = 13). Finally, we included 607 patients of whom 66 received sildenafil add-on therapy to antifibrotic agents, either pirfenidone or nintedanib (Fig. 1). To evaluate the effect of sildenafil, patients who received antifibrotic treatment with sildenafil (sildenafil group) and those who received antifibrotic treatment alone (no-sildenafil group) were compared. The index date was defined as the first occurrence of the relevant treatment: concurrent sildenafil and antifibrotic treatment in the sildenafil group; and antifibrotic treatment in the no-sildenafil group. In order to balance the baseline characteristics, propensity score matching was performed to generate matched pairs for the sildenafil and no-sildenafil groups.

All patients fulfilled the IPF diagnostic criteria of the American Thoracic Society (ATS), European Respiratory Society (ERS), Japanese Respiratory Society, and Latin American Thoracic Association^1^. The diagnosis of each patient was made through a multidisciplinary discussion. The decision to start sildenafil was at the discretion of the treating physician, based on each patient’s symptoms and the presence of pulmonary hypertension. Sildenafil was given at a dose of 20 mg three times daily. Patients’ follow-up data were collected from the index date to 31 July 2019. This study was approved by the Institutional Review Board of Asan Medical Center (No.: 2021–0041), and the requirement for informed consent was waived due to the retrospective nature of the study by the Institutional Review Board of Asan Medical Center. All methods were performed in accordance with the relevant guidelines and regulations of the journal.

### Study data and outcomes

The outcomes of this study included the annual decline rate of FVC, risks of all-cause mortality, hospitalization (all-cause and respiratory-related), and acute exacerbation. Spirometric parameters^14^, DL_CO_^15,16^_,_ and total lung capacity^17^ were measured according to the ATS/ERS recommendations. Data regarding the patients’ baseline characteristics and clinical outcomes including death, hospitalization, and development of acute exacerbation were retrospectively obtained from electronic medical records, telephone interviews, and/or the records of the National Health Insurance of Korea. Respiratory-related hospitalization was defined as an unscheduled hospitalization resulting from acute respiratory worsening such as pneumonia, pneumothorax, and acute exacerbation. Acute exacerbation was defined using the criteria proposed by Collard et al. in 2016^18^.

Echocardiographic evidence of pulmonary hypertension was defined as the presence of at least one of the following: (1) RV dysfunction; (2) RV enlargement; (3) maximal tricuspid regurgitation velocity (TR V_max_) > 3.4 m/s; or estimated systolic pulmonary arterial pressure > 40 mmHg.

### Statistical analysis

Patient baseline clinical characteristics and clinical outcomes were summarized by treatment arm using the mean ± standard deviation for continuous variables or percentages for categorical variables. Student’s t-test was used for continuous data, and Pearson’s chi-squared test or Fisher’s exact test was used for categorical data.

Propensity score matching was performed to adjust for differences in baseline characteristics between the sildenafil and no-sildenafil groups. The matched variables were age, sex, body mass index, FVC, and DL_CO_. The annual decline rate of FVC was estimated using a linear mixed model with random effect for each patient and their matching pair. The relative risks of mortality, hospitalization, and acute exacerbation were analyzed using a Cox proportional hazards model. To control the bias that patients were more likely to receive sildenafil when they were found to have pulmonary hypertension, which is a poor prognostic factor in itself^7,19^, the multivariable analyses were adjusted for the presence of pulmonary hypertension and the Charlson Comorbidity Index. All statistical analyses were performed using SPSS software (version 22.0; IBM Corporation, Somers, NY, USA) or R version 3.3.3 (R Foundation for Statistical Computing, Vienna, Austria).

## Results

### Baseline characteristics

Among the 607 patients treated with an antifibrotic agent, 66 also received sildenafil. The clinical characteristics of the patients in each group are shown in Supplementary Table S1. Briefly, patients who received sildenafil were significantly older and exhibited lower FVC, forced expiratory volume in 1 s, DL_CO_, and total lung capacity, a shorter 6-min walk distance, and a lower oxygen saturation nadir during a 6-min walk test.

Fifty-one matched pairs were created by propensity score matching. Their baseline clinical characteristics are shown in Table 1. Among these 102 patients, the mean age was 69.5 years and 80.4% were male; mean FVC and DL_CO_ were 51.7% and 29.5% of the predicted values, respectively. The median time from IPF diagnosis to the initiation of antifibrotic treatment in all matched patients was 13.0 months (sildenafil group: 12.0 months vs. no-sildenafil group: 15.0 months; *p* = 0.638). In the sildenafil group, the median interval between the initiation of the antifibrotic treatment and that of the sildenafil treatment was 9.0 months.

**Table 1:** Comparison of baseline characteristics between the sildenafil and no-sildenafil matched pair groups among patients with idiopathic pulmonary fibrosis.

	Sildenafil group	No-sildenafil group	*P* value
Number of study patients	51	51	
Age (years)	69.6 ± 7.8	69.5 ± 7.7	0.929
Male	41 (80.4)	41 (80.4)	> 0.999
BMI	24.4 ± 2.8	24.2 ± 3.4	0.694
Smoking status			0.973
Current	5 (9.8)	5 (9.8)	
Ex-smoker	34 (66.7)	33 (64.7)	
Non-smoker	12 (23.5)	13 (25.5)	
Charlson Comorbidity Index score*	1.6 ± 0.8	1.9 ± 1.0	0.033
Pulmonary hypertension in Echocardiography	25 (49.0)	6 (11.8)	< 0.001
PFT (% of the predicted value)			
FVC	52.0 ± 12.8	51.3 ± 14.7	0.797
FEV_1_	64.9 ± 17.0	62.9 ± 16.9	0.544
FEV_1_/FVC	88.8 ± 9.4	85.9 ± 13.0	0.205
DL_CO_	29.3 ± 11.6	29.6 ± 11.6	0.914
TLC	53.6 ± 10.0	54.1 ± 14.3	0.835
6MWD (m)	325.0 ± 132.1	300.5 ± 122.3	0.341
6MWT minimum saturation (%)	84.2 ± 5.0	84.5 ± 5.7	0.829
Antifibrotic agent			0.004
Pirfenidone	20 (39.2)	34 (66.7)	
Nintedanib	31 (60.8)	15 (29.4)	
Pirfenidone → nintedanib	0 (0.0)	2 (3.9)	

Data are presented as mean ± standard deviation or number (%).

*Comorbidities in each group are described in Supplementary Table S2.

BMI, body mass index; PFT, pulmonary function test; FVC, forced vital capacity; FEV_1_, forced expiratory volume in 1 s; DL_CO_, diffusing capacity of the lung for carbon monoxide; TLC, total lung capacity; 6MWD, 6-min walk distance; 6MWT, 6-min walk test.

The median follow-up duration from the index date was 7.0 months (sildenafil: 7.0 months vs. no-sildenafil group: 8.0 months; *p* = 0.139). In the sildenafil group, nintedanib was more commonly used than pirfenidone (60.8% vs. 39.2%), in contrast to that in the no-sildenafil group (29.4% vs. 66.7%). The no-sildenafil group showed a significantly higher mean Charlson Comorbidity Index score than the sildenafil group (*p* = 0.033).

### Annual FVC decline rate

**Figure 2:**
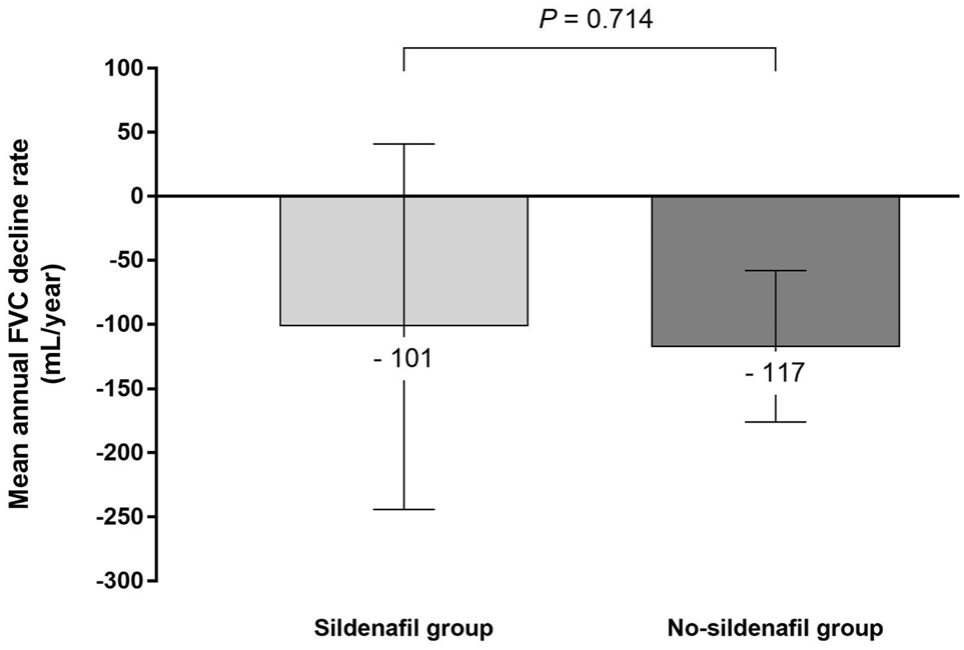
Comparison of the estimated annual FVC decline rate between the sildenafil and no-sildenafil groups among IPF patients with antifibrotic treatment. The annual decline rate of FVC was estimated using a linear mixed model. The difference in the annual FVC decline rates between the groups was not statistically significant (− 101 mL/year in the sildenafil group vs. − 117 mL/year in the no-sildenafil group; *p* = 0.714). FVC, forced vital capacity; IPF, idiopathic pulmonary fibrosis.

The estimated annual FVC decline rates in the sildenafil and no-sildenafil groups are shown in Fig. 2. The FVC decline rates of − 101 mL/year (95% confidence interval CI − 244 to 41 mL) in the sildenafil group and − 117 mL/year (95% CI − 176 to − 58 mL) in the no-sildenafil group did not differ significantly (*p* = 0.714).

### Clinical outcomes

The number of patients who experienced at least one of the clinical outcomes is shown in Table 2. A total of 89 patients (87.3%) died between the index date and the end of study. The median survival was 12.0 months (95% CI 8.7–15.3 months) in all patients. There was no significant difference in all-cause mortality between the sildenafil and no-sildenafil groups (88.2% vs. 86.3%, respectively; *p* > 0.999). Most of the hospitalizations were due to respiratory-related causes. Only one patient, a member of the sildenafil group, was hospitalised for a non-respiratory-related cause. A significantly greater number of patients experienced all-cause hospitalization in the sildenafil group (54.9% vs. 33.3%; *p* = 0.046). A higher number of acute exacerbations occurred in the sildenafil group than the no-sildenafil group, but the difference was not statistically significant (31.4% vs. 15.7%, respectively; *p* = 0.101).

**Table 2:** Comparison of clinical outcomes for patients with idiopathic pulmonary fibrosis between the sildenafil and no-sildenafil groups.

	Sildenafil group	No-sildenafil group	*P* value
All-cause mortality	41 (80.4)	37 (72.5)	0.484
All-cause hospitalization	28 (54.9)	17 (33.3)	0.046
Respiratory-related hospitalization	27 (52.9)	17 (33.3)	0.046
Non-respiratory-related hospitalization	1 (2.0)	0 (0.0)	> 0.999
Acute exacerbation	16 (31.4)	8 (15.7)	0.101

Data are presented as number (%).

The median follow-up durations were 7.0 and 8.0 months in the sildenafil and no-sildenafil groups, respectively (*p* = 0.139).

### Effect of sildenafil on the risk of death, hospitalization, and acute exacerbation

The effect of sildenafil was analyzed using the Cox proportional hazards model adjusted for the Charlson Comorbidity Index score and the presence of pulmonary hypertension (Table 3). Sildenafil did not appear to have a significant effect on all-cause mortality, all-cause hospitalization, respiratory-related hospitalization, or acute exacerbation.

**Table 3:** Impact of sildenafil on the risks of clinical outcomes in patients with idiopathic pulmonary fibrosis under antifibrotic treatment^*^.

	Hazard ratio	95% confidence interval	*P* value
All-cause mortality	1.395	0.748–2.602	0.296
All-cause hospitalization	2.107	0.934–4.752	0.073
Respiratory-related hospitalization	2.051	0.924–4.551	0.077
Acute exacerbation	2.144	0.696–6.608	0.184

*Assessed by multivariable Cox analyses adjusted for the presence of pulmonary hypertension and Charlson comorbidity index.

## Discussion

Although the management of IPF has evolved with the introduction of antifibrotic treatment, it is widely recognized that the efficacy of antifibrotic agents is limited and that there is a need for additional therapies. In this retrospective study, the effect of sildenafil was evaluated in IPF patients receiving antifibrotic treatment. Sildenafil add-on therapy demonstrated no significant benefits with respect to FVC decline rate, all-cause mortality, hospitalization, and acute exacerbation.

Previous randomized controlled trials on sildenafil included patients with advanced IPF as represented by a DL_CO_ below 35% or 40% of the predicted value^9,11,12^. As in other chronic lung diseases, pulmonary hypertension often accompanies the disease course of IPF^6^. It has been reported that the prevalence of pulmonary hypertension increases with the severity of underlying IPF, ranging from 8 to 15% at initial diagnosis^20,21^ to 30–50% in the advanced stages^7,22,23^. In one retrospective study, which included 44 patients with IPF who underwent lung transplantation, it was revealed that 86.4% of the patients had pulmonary hypertension at the time of the lung transplantation^24^. The majority of the patients in our study had advanced-stage IPF, with mean DL_CO_ of 29.3% and 29.6% in the sildenafil and no-sildenafil groups, respectively. Because sildenafil tends to be prescribed to advanced-IPF patients in real clinical practice, propensity score matching generated a cohort of patients with advanced IPF in our study.

Sildenafil was not associated with a reduced rate of FVC decline. Although the number of patients included in our study was small, it is notable that the FVC decline rate in the no-sildenafil group (antifibrotic treatment alone) was similar to those seen under antifibrotic treatments in previous clinical trials^3,11,25^. In our study, both the sildenafil and no-sildenafil groups had similar estimated annual FVC decline rates. However, previous clinical trials showed contrasting FVC decline rate results. The INSTAGE trial evaluated 273 patients with advanced IPF, defined as a DL_CO_ < 35% predicted; treatment with sildenafil plus nintedanib resulted in a smaller decline in FVC than nintedanib treatment alone (− 20.8 vs. − 58.2 mL over 24 weeks)^12^. In another study, the effect of pirfenidone plus sildenafil was compared with pirfenidone alone in 177 IPF patients with a DL_CO_ ≤ 40% predicted and a risk of pulmonary hypertension. The FVC change from baseline was greater under combination treatment than under pirfenidone alone (− 145.0 vs. − 93.0 mL)^11^. Although direct comparison of these results may not be appropriate because of the different inclusion criteria in the studies, one may question if nintedanib and pirfenidone synergize differently with sildenafil. However, there is a lack of evidence that sildenafil interacts differently with the two anti-fibrotic medications and so far, pirfenidone and nintedanib are considered to exert similar beneficial effects in patients with IPF^2,3,26^. Further studies may help to determine whether the effect of sildenafil varies with the concurrent antifibrotic medication.

It has been assumed that patients may benefit from treatment of the pulmonary hypertension accompanying IPF. Interestingly, however, previous studies with endothelin receptor antagonists failed to show clinical benefits in IPF patients^27–29^. In particular, ambrisentan, a selective endothelin A receptor antagonist approved for the treatment of idiopathic pulmonary arterial hypertension, resulted in higher mortality and hospitalization rates in IPF patients^28^. Direct extrapolation of the ambrisentan study results to sildenafil should be avoided, because sildenafil has a different mechanism of action. However, consideration of the reasons that endothelin receptor antagonists were ineffective^27,29^ or even harmful^28^ may provide guidance in interpreting the results of sildenafil studies. The authors of one study suggested that the poorer outcomes under ambrisentan treatment could be due to the impairment of appropriate vascular responses during acute respiratory stress or exacerbation following the modulation of vascular remodeling by ambrisentan^28^. It is unclear whether this hypothesis can explain why sildenafil produced no significant effects in our study; further studies are needed to elucidate how sildenafil functions in patients with IPF.

Patients with pulmonary hypertension have been shown to have poorer exercise capacity^7,30^ and higher mortality^7,19,31^ than those without pulmonary hypertension. In our study, 76.5% of the patients died by the end of the study, the median survival being 12.0 months. It is well known that disease progression is the most common cause of death in IPF^19,32^. Acute exacerbation is also frequently fatal^33^. In our study, the number of patients who experienced respiratory-related hospitalization or acute exacerbation was lower than the number of patients who died. Other possible causes contributing to mortality include right heart failure, given the severity of the disease in the study patients. In a previous study, sildenafil appeared to be more effective in preserving exercise capacity and improving health-related quality of life in patients with RV dysfunction than in those without^10^. However, this benefit was not reproduced in the subgroup analysis of the INSTAGE trial involving patients with RV dysfunction^13^. In our study, patients in the no-sildenafil group experienced fewer respiratory-related hospitalizations, so more deaths resulting from right heart failure may have occurred. The effects of sildenafil on hemodynamics, and ultimately mortality, in patients both with and without RV dysfunction, need to be further researched.

There are some limitations that should be addressed. First, this was a retrospective study conducted at a single center. To balance the baseline patient differences, propensity matching was performed. It needs to be noted that pulmonary hypertension was more frequently found in the sildenafil group after matching. Pulmonary hypertension could not be included as a matching variable because it resulted in only small number of patients in each group. Instead, multivariable analyses were adjusted for the Charlson Comorbidity Index score and the presence of pulmonary hypertension on echocardiography to eliminate the potential biases of a non-randomized study. Although we attempted to overcome the limitation by applying various statistical adjustments, it should be considered that confounding might have influenced the outcomes when interpreting the results of our study. Second, the proportion of patients receiving pirfenidone and nintedanib differed in the sildenafil and no-sildenafil groups. Nintedanib predominated in the sildenafil group, whereas pirfenidone predominated in the no-sildenafil group. It is hard to determine whether this factor influenced the study result due to the lack of evidence. However, so far, pirfenidone and nintedanib treatments are considered to result in similar clinical outcomes; both medications have been demonstrated to reduce the FVC decline rate by approximately 50% compared with the control group^2,3,34^. In addition, one study that used a large U.S. insurance database showed that all-cause mortality was not significantly different between patients treated with pirfenidone and nintedanib^26^. Third, the study patients were collected over 14 years during which therapeutic approach has changed. In patients who were diagnosed with IPF before the antifibrotic era, there was a delay between the diagnosis and initiation of antifibrotic treatment. This might have influenced the clinical outcomes of the patients. However, the median time from IPF diagnosis to the initiation of antifibrotic treatment was not significantly different between the groups. Fourth, we defined the presence of pulmonary hypertension using echocardiographic findings. Although echocardiography is a useful and important component of patient management, its performance may not be reliable in patients with advanced lung disease^7,35^. The gold standard for the diagnosis of pulmonary hypertension is right heart catheterisation, but this test is invasive and not possible in all patients^8^.

In conclusion, sildenafil add-on treatment had no significant effects on patients’ clinical outcomes, including FVC decline rate, all-cause mortality, hospitalization, and acute exacerbation, in patients with IPF.

## Supplementary Information

Supplementary Information.

## Data Availability

The datasets generated during and/or analyzed during the current study are available from the corresponding author on reasonable request.
