# Supplementary material for: Effect of sildenafil added to antifibrotic treatment in idiopathic pulmonary fibrosis
Source: Sci Rep. 2021 Sep 8;11:17824. doi: 10.1038/s41598-021-97396-z (PMC8426395; doi:10.1038/s41598-021-97396-z)
Supplement: Supplementary file 1 — Supplementary Information. [file 41598_2021_97396_MOESM1_ESM.docx]

**Supplementary Data**

**Appendix to: Effect of Sildenafil added to Antifibrotic Treatment in Idiopathic Pulmonary Fibrosis**

Jieun Kang^1^, Jin Woo Song^2^

^1^Division of Pulmonary and Critical Care Medicine, Department of Internal Medicine, Ilsan Paik Hospital, Inje University College of Medicine, Goyang, Republic of Korea

^2^Department of Pulmonary and Critical Care Medicine, Asan Medical Center, University of Ulsan College of Medicine, Seoul, Republic of Korea

Corresponding author: Jin Woo Song, MD, PhD

Department of Pulmonary and Critical Care Medicine, Asan Medical Center, University of Ulsan College of Medicine, 88 Olympic-Ro 43-gil, Songpa-gu, Seoul 05505, Republic of Korea

Tel.: +82-2-3010-3993

Fax: +82-2-3010-6968

E-mail: [jwsongasan@gmail.com](mailto:jwsongasan@gmail.com)

**Supplementary Table S1. Comparison of the baseline characteristics of 607 idiopathic pulmonary fibrosis patients in the sildenafil and no-sildenafil groups before propensity score matching**

|  | Sildenafil group | No-sildenafil group | p-value |
| --- | --- | --- | --- |
| Number of study patients | 66 | 541 |  |
| Age (years) | 69.7 ± 7.5 | 65.5 ± 7.8 | <0.001 |
| Male | 52 (78.8) | 451 (83.4) | 0.386 |
| BMI | 24.2 ± 2.9 | 25.0 ± 3.0 | 0.053 |
| Smoking status |  |  | 0.497 |
| Current | 5 (7.6) | 68 (12.6) |  |
| Ex-smoker | 44 (66.7) | 344 (63.6) |  |
| Non-smoker | 17 (25.8) | 129 (23.8) |  |
| Charlson Comorbidity Index score | 1.6 ± 0.9 | 1.8 ± 1.0 | 0.156 |
| PFT (% of the predicted value) |  |  |  |
| FVC | 52.1 ± 13.3 | 66.2 ± 13.7 | <0.001 |
| FEV_1_ | 64.9 ± 16.6 | 75.6 ± 14.5 | <0.001 |
| FEV_1_/FVC | 101.7 ± 19.0 | 81.9 ± 8.0 | <0.001 |
| DL_CO_ | 26.2 ± 11.5 | 52.8 ± 15.3 | <0.001 |
| TLC | 54.4 ± 10.0 | 66.7 ± 11.7 | <0.001 |
| 6MWD (m) | 313.4 ± 12.92 | 424.9 ± 98.0 | <0.001 |
| 6MWT minimum saturation (%) | 84.1 ± 5.2 | 90.0 ± 4.8 | <0.001 |
| Antifibrotic agent |  |  | <0.001 |
| Pirfenidone | 39 (59.1) | 443 (81.9) |  |
| Nintedanib | 27 (40.9) | 57 (10.5) |  |
| Pirfenidone 🡪 nintedanib | 0 (0.0) | 32 (5.9) |  |
| Nintedanib 🡪 pirfenidone | 0 (0.0) | 9 (1.7) |  |

Data are presented as mean ± standard deviation or number (%).

BMI, body mass index; PFT, pulmonary function test; FVC, forced vital capacity; FEV_1_, forced expiratory volume in 1 s; DL_CO_, diffusing capacity of the lung for carbon monoxide; TLC, total lung capacity; 6MWD, 6-min walk distance; 6MWT, 6-min walk test.

**Supplementary Table S2. Comorbidities in in the sildenafil and no-sildenafil groups**

|  | Sildenafil group | No-sildenafil group | p-value |
| --- | --- | --- | --- |
| Number of study patients | 51 | 51 |  |
| DM without complications | 16 (31.4) | 13 (25.5) | 0.661 |
| DM with complications | 0 (0.0) | 2 (3.9) | >0.999 |
| Myocardial infarction | 4 (7.8) | 5 (9.8) | >0.999 |
| Congestive heart failure | 1 (2.0) | 2 (3.9) | >0.999 |
| Stroke or transient ischemic attack | 1 (2.0) | 6 (11.8) | 0.112 |
| Peptic ulcer disease | 1 (2.0) | 0 (0.0) | >0.999 |
| Chronic liver disease | 1 (2.0) | 1 (2.0) | >0.999 |
| Dementia | 0 (0.0) | 0 (0.0) | n.a |
| Peptic ulcer disease | 1 (2.0) | 0 (0.0) | >0.999 |
| Solid tumor |  |  | 0.017 |
| Localized | 3 (5.9) | 9 (17.6) |  |
| Metastatic | 0 (0.0) | 4 (7.8) |  |
| Leukemia or lymphoma | 0 (0.0) | 0 (0.0) | n.a |

Data are presented as mean ± standard deviation or number (%).

DM, diabetes mellitus.
